# Efficacy of Thymoquinone and Hesperidin in Attenuating Cardiotoxicity from 5-Fluorouracil: Insights from In Vivo and In Silico Studies

**DOI:** 10.3390/toxics12090688

**Published:** 2024-09-23

**Authors:** Juveriya Farooq, Rokeya Sultana, Jainey P. James, Zakiya Fathima C, Ali F. Almutairy, Abubakar Siddique Mustafa Hussain

**Affiliations:** 1 Department of Pharmacology, Yenepoya Pharmacy College & Research Centre, Yenepoya (Deemed to be University), Mangalore 575018, India; 2 Department of Pharmacognosy, Yenepoya Pharmacy College & Research Centre, Yenepoya (Deemed to be University), Mangalore 575018, India; 3 Department of Pharmaceutical Chemistry, NGSM Institute of Pharmaceutical Sciences (NGSMIPS), Nitte (Deemed to be University), Mangalore 575018, India; jaineyjames@nitte.edu.in (J.P.J.); zakiyafathimaksd@gmail.com (Z.F.C.); 4 Department of Pharmacology and Toxicology, College of Pharmacy, Qassim University, Buraydah 51452, Saudi Arabia; a.almotairi@qu.edu.sa; 5 Discipline of Clinical Pharmacy, School of Pharmaceutical Sciences, Universiti Sains Malaysia, Gelugor 11700, Malaysia; getabu2u@gmail.com

**Keywords:** 5-Fluorouracil, cardiotoxicity, thymoquinone, hesperidin, molecular docking

## Abstract

5-Fluorouracil (5-FU) is widely used in chemotherapy but poses serious risks of cardiotoxicity, which can significantly affect treatment outcomes. Identifying interventions that can prevent these adverse effects without undermining anticancer efficacy is crucial. This study investigates the efficacy of Thymoquinone (TQ) and Hesperidin (HESP) in preventing cardiotoxicity induced by 5-FU in Wistar rats and elucidates the molecular interactions through docking studies. We employed an experimental design involving multiple groups of Wistar rats exposed to 5-FU, with and without the concurrent administration of TQ and HESP. Cardiac function markers, oxidative stress indicators, and inflammatory markers were assessed. Additionally, molecular docking was used to analyze the interaction of TQ and HESP with key inflammatory proteins. Treatment with TQ and HESP not only lowered levels of cardiac enzymes but also improved antioxidant capacity and reduced inflammation in cardiac tissues. Notably, the combination of TQ and HESP provided more significant protective effects than either agent alone. Molecular docking supported these findings, showing effective binding of TQ and HESP to inflammatory targets. TQ and HESP demonstrate potential as protective agents against cardiotoxicity in 5-FU-treated rats, with their combined use offering enhanced protection. These findings suggest a viable strategy for reducing cardiac risks associated with 5-FU chemotherapy.

## 1. Introduction

5-Fluorouracil (5-FU) is widely utilized as a chemotherapeutic drug for various malignancies, including colorectal, breast, head and neck, and gastric cancers [1]. Its primary mechanism involves inhibiting thymidylate synthase, thereby disrupting DNA synthesis and inducing apoptosis [2]. Despite its widespread use and clinical efficacy, the administration of 5-FU is associated with a spectrum of adverse effects, among which cardiotoxicity is particularly concerning due to its potential severity and impact on patient outcomes [3]. 5-FU can induce cardiotoxicity, presenting as vasospasm, angina, myocardial infarction, blood pressure changes, and cardiogenic shock, with a reported incidence of 2.16% in chemotherapy patients [4,5]. Factors that increase the risk of cardiotoxicity include prolonged infusion rates, existing coronary artery disease (CAD), prior heart conditions, chronic kidney disease (CKD), and concurrent use of cisplatin. However, cardiotoxicity can also occur in patients without pre-existing heart conditions or additional chemotherapy agents [6,7]. Beyond the immediate cardiovascular risks, 5-FU induced cardiac toxicity has led to early cessation of treatment, potentially resulting in inadequate cancer treatment and negatively affecting survival [8].

Plant-derived natural products, such as alkaloids, flavonoids, and terpenoids, show promise in preventing anticancer drug-induced toxicity due to their safer profiles compared to synthetic drugs [9]. Phytopharmaceuticals have been studied extensively for their ability to mitigate chemotherapy-induced side effects like gastrointestinal toxicity, nephrotoxicity, cardiotoxicity, and neurotoxicity [10]. Thymoquinone (TQ), a monoterpene molecule characterized as 2-methyl-5-isopropyl-1,4-benzoquinone, is sourced from the seeds of *Nigella sativa* L., a plant in the Ranunculaceae family [11]. This compound is renowned for a broad spectrum of pharmacological effects, including its ability to act as an anti-inflammatory, antimicrobial, antioxidant, antiasthmatic, antihypertensive, and anticancer agent [12,13]. Thymoquinone also plays a crucial role in shielding against the side effects of anticancer medications by targeting antioxidant enzymes, anti-inflammatory cytokines, and proteins involved in apoptosis, thereby alleviating oxidative stress, inflammation, and cell death in various tissues [14,15].

Hesperidin (HESP), identified as 3,5,7-trihydroxyflavanone 7-rhamnoglucoside, is a flavanone found in citrus fruits [16]. This compound has undergone extensive research for its health benefits and pharmacological properties. HESP is used in the management of various conditions, including type 2 diabetes, cancer, cardiovascular diseases, and neurological and psychiatric disorders [17]. Furthermore, it has demonstrated potential in enhancing immune function, reducing inflammation, and offering protection against oxidative stress [18,19].

This study introduces the novel combination of TQ and HESP to address a critical gap in the management of 5-FU-induced cardiotoxicity. While synthetic cardioprotective agents have been studied, they often present adverse effects [20,21,22]. In contrast, TQ and HESP, both of natural origin, offer a less toxic, yet equally potent, alternative. The concurrent administration of TQ and HESP might offer a synergistic protective effect against cardiotoxicity induced by 5-FU by influencing various pathways that contribute to these adverse effects. This study is designed to thoroughly explore the protective benefits of TQ and HESP, both individually and combined, against cardiotoxicity caused by 5-FU in vivo. Additionally, molecular docking studies were conducted to understand the binding interactions of TQ and HESP with key inflammatory targets involved in 5-FU-induced cardiotoxicity.

## 2. Materials and Methods

### 2.1. Materials

5-FU, TQ, and HESP were obtained from Sigma, St. Louis, MO, USA.

### 2.2. Molecular Docking

#### 2.2.1. Software

The software used for molecular docking is Schrodinger 2018-3 suite device Maestro 11.7.012. Structure of the ligands (TQ, HESP) and SMILES was taken from Pub-chem.

#### 2.2.2. Ligand Preparation

Ligands were neutralized and desalted, and tautomer generation was prevented to maintain specific chirality using the Ligprep tool (Schrodinger LLC, New York, NY, USA).

#### 2.2.3. Protein Preparation

The crystalline structures in both two and three dimensions of three targeted proteins were obtained from the Protein Data Bank (http://www.rcsb.org/) (accessed on 2 June 2024). The proteins extracted included IL-6 (PDB ID: 1N26), IL-8 (PDB ID: 4XDX), and TNF-α (PDB ID: 2AZ5). These were processed using the Protein Preparation Wizard in Schrodinger software. During this preprocessing stage, bond orders were established, hydrogens added, and zero-order bonds to metals along with disulfide bonds were formed. Furthermore, missing side chains and loops were reconstructed using the Prime module, and water molecules more than 5 Å away from hetero groups were eliminated. The ligand states were stabilized within a pH range of 7 ± 2 to maintain hetero states.

#### 2.2.4. Receptor Grid Generation

A grid defining the 3D co-ordinates (X, Y, Z) for ligand binding was created for the minimized protein using the Receptor Grid Generation tool provided by Schrodinger LLC, based in New York, NY, USA.

#### 2.2.5. Ligand Docking

Ligand docking was conducted with the Glide-XP tool from Schrodinger LLC, New York, NY, USA. The receptor grid previously generated was uploaded, and the prepared ligands were loaded into the workspace as out.maegz files.

### 2.3. Animal Studies

#### 2.3.1. Experimental Animals

Male Wistar albino rats, with individual weights ranging from 170 to 200 g, were sourced from the animal facility at Yenepoya University, Mangaluru, India, following institutional animal ethics committee protocols (Clearance No: YU/IAEC/13/2022).

#### 2.3.2. Experimental Design

Rats were divided into seven distinct groups, with each group consisting of six rats. Group 1, the control group, received saline orally for eight days. Group 2 was given saline orally for the same duration, followed by a single intraperitoneal (I.P) injection of 5-FU (150 mg/kg body weight) on the 5th day [23]. Group 3 received TQ at a dose of 100 mg/kg orally for eight days [24], while Group 4 was administered HESP at the same dose and route [25]. Group 5 combined TQ treatment (100 mg/kg for eight days) with a single I.P injection of 5-FU (150 mg/kg) on the fifth day. Group 6 followed a similar regimen with HESP (100 mg/kg for eight days) and a single dose of 5-FU on the fifth day. Finally, Group 7 was treated with both HESP and TQ (50 mg/kg each, orally for eight days), followed by a single I.P injection of 5-FU (150 mg/kg) on the fifth day.

#### 2.3.3. Sample Collection and Storage

Blood samples were collected 24 h after the final treatment dose under anesthesia, and rats were euthanized using ketamine hydrochloride and xylazine (75 + 20 mg/kg). Serum was separated by centrifuging the samples at 3000 rpm and then stored at a temperature of −80 °C. Cardiac tissues were also frozen at −80 °C for further analysis.

#### 2.3.4. Measurement of Serum Cardiac Function Markers

The levels of creatine kinase-MB (CK-MB), creatine kinase-NAC (CK-NAC), and lactate dehydrogenase (LDH) were determined using commercially availablekits obtained from Accurex Biomedical (Mumbai, India) following the protocols provided by the manufacturer.

#### 2.3.5. Measurement of Tissue Oxidative Stress Markers

Markers indicative of oxidative stress such as malondialdehyde (MDA) and myeloperoxidase (MPO), as well as antioxidant markers such as superoxide dismutase (SOD), glutathione (GSH), and catalase (CAT) were quantified in cardiac tissues using Quantikine ELISA kits (R&D Systems Inc., Minneapolis, MN, USA) in accordance with the instructions provided by the manufacturer.

#### 2.3.6. Measurement of Inflammatory Biomarkers

Inflammatory markers, that is, interleukin-6 (IL-6), interleukin-8 (IL-8), and tumor necrosis factor-α (TNF-α), were measured in cardiac tissues using Quantikine ELISA kits (R&D Systems Inc., USA), adhering to the manufacturer’s guidelines.

#### 2.3.7. Histopathological Examination

Cardiac tissues were preserved in 10% formalin, subsequently embedded in paraffin, sectioned longitudinally, and stained with hematoxylin and eosin for detailed microscopic analysis.

### 2.4. Statistical Analysis

Data are expressed as mean ± SD. Group comparisons were conducted using one-way ANOVA, followed by the Tukey-Kramer post-hoc analysis. A *p* value of less than 0.05 was considered statistically significant. The analyses were executed using GraphPad Prism, version 8.

## 3. Results

### 3.1. Molecular Interactions and Docking Score

A docking study was conducted to identify the exact binding sites on different inflammatory targets. In this research, docking was performed on the active sites of three target proteins: 1N26, 4XDX, and 2AZ5, with TQ and HESP.

**Figure 1:**
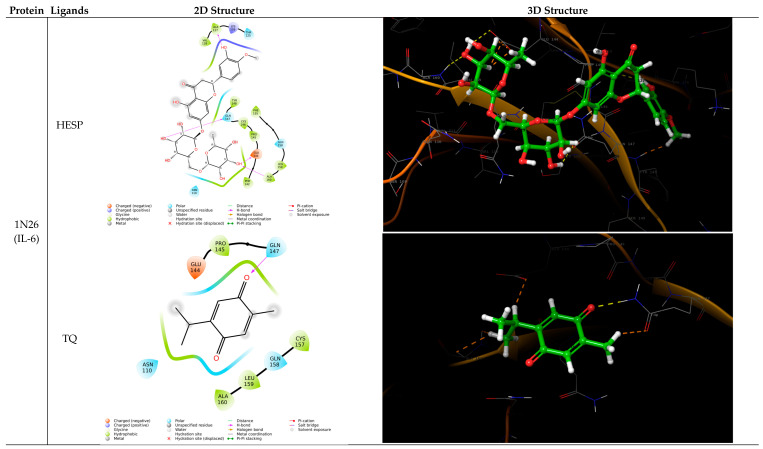

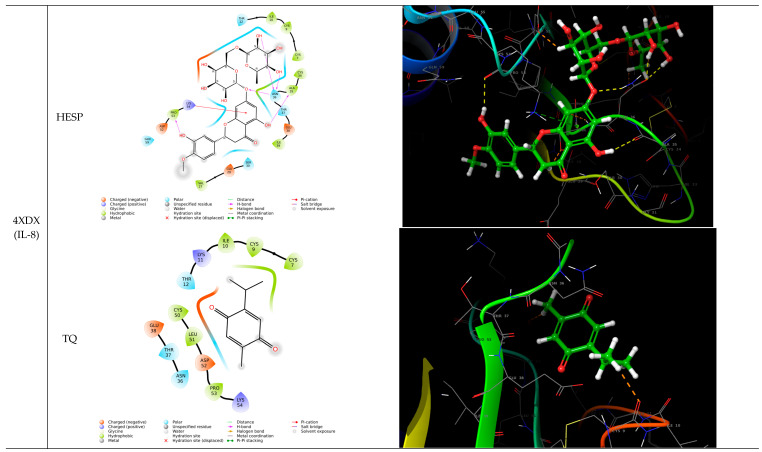

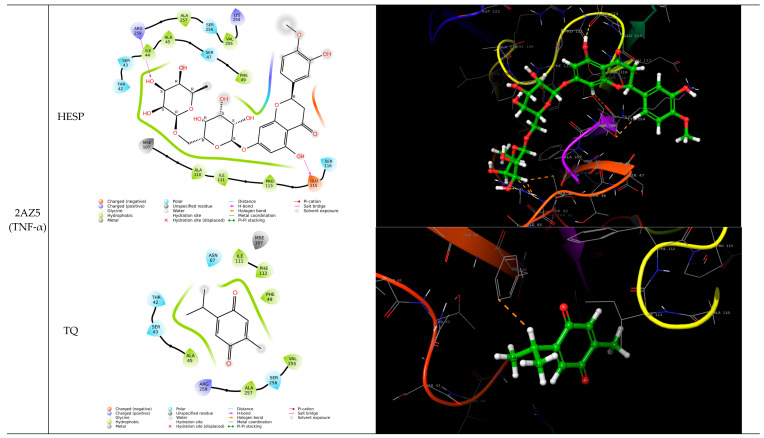
2D and 3D conformations of Hesperidin and Thymoquinone with 1N26, 4XDX, and 2AZ5.

Detailed analysis of the results showed that HESP has better binding with target proteins than TQ. The docking scores and ligand interactions are tabulated in Table 1; 2D and 3D conformations are reported in Figure 1.

### 3.2. In Vivo Studies

#### 3.2.1. Effect of TQ and HESP on Cardiac Marker Enzymes in Treated Rats

**Figure 2:**
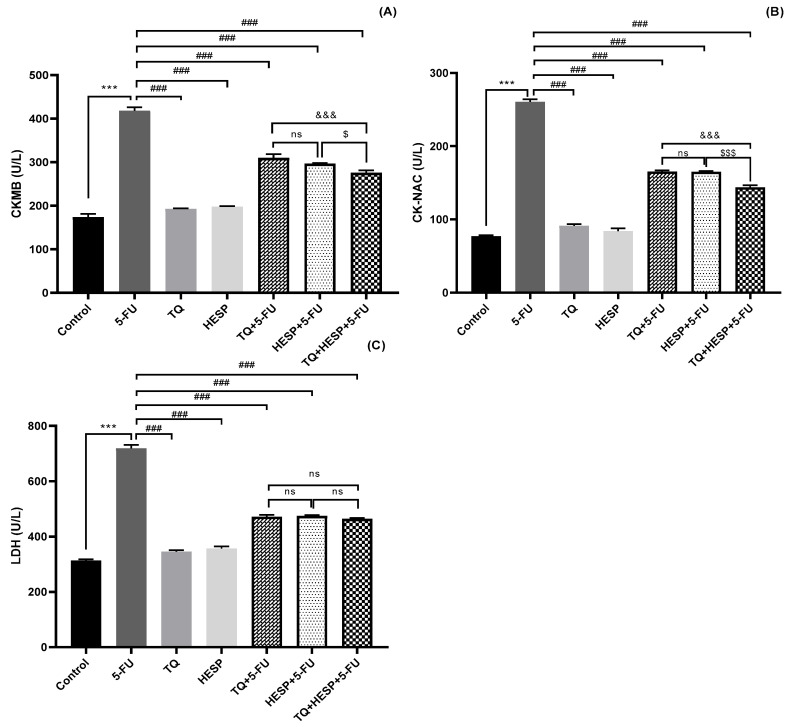
The effect of TQ, HESP, and 5-FU administration on the level of cardiac enzymes, and comparison of different groups with each other (one-way Anova followed by Tukey-Kramer multiple comparison test). (**A**) CKMB level; (**B**) CK-NAC level; (**C**) LDH level. All results are expressed as the mean ± SD. *** *p* < 0.001 vs. control group; ### *p* < 0.001 vs. 5-FU group; &&& *p* < 0.001 vs. TQ+5-FU group; $ *p* < 0.05, $$$ *p* < 0.001 vs. HESP+5-FU group. ns = non-significant; CKMB = creatinine kinase myoglobin binding; CK-NAC = N-acetyl-cysteine activated creatinine kinase; LDH = lactate dehydrogenase; TQ = Thymoquinone; HESP= Hesperidin; 5-FU = 5-Fluorouracil.

The results showed that CK-MB, CK-NAC, and LDH levels were significantly increased in the 5-FU-treated group as compared to the control group (*** *p* < 0.001). Concomitant administration of TQ, HESP, and their combination with 5-FU led to a significant decrease in CK-MB, CK-NAC, and LDH levels compared to the group treated with 5-FU alone. Additionally, among the groups treated with TQ+5-FU, HESP+5-FU, and their combination with 5-FU, the combination group demonstrated a significant improvement. Furthermore, when comparing between the TQ+5-FU and HESP+5-FU groups, the HESP+5-FU group displayed superior outcomes (Figure 2A–C).

#### 3.2.2. Effect of TQ and HESP on Tissue Antioxidants/Oxidative Stress Marker in Treated Rats

**Figure 3:**
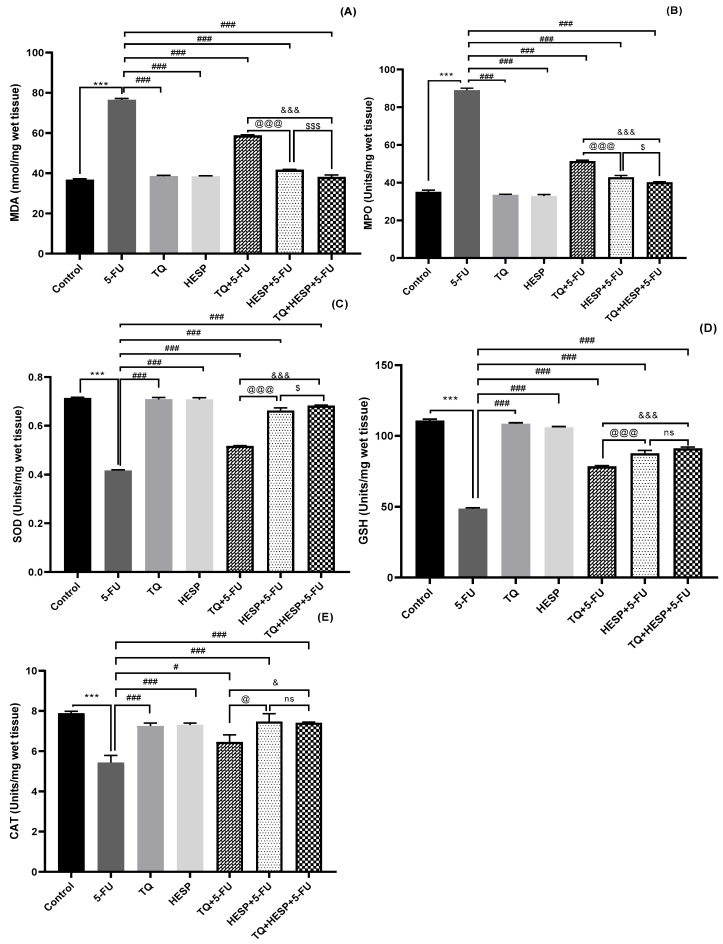
The effect of TQ, HESP, and 5-FU administration on cardiac tissue oxidative stress parameters and comparison of different groups with each other (one-way Anova followed by Tukey-Kramer multiple comparison test). (**A**) MDA level; (**B**) MPO level; (**C**) SOD level; (**D**) GSH level; (**E**) CAT level. All results are expressed as the mean ± SD. ns = non-significant; *** *p* < 0.001 vs. control group; ### *p* < 0.001, # *p* < 0.05 vs. 5-FU group; @ *p* < 0.05, @@@ *p* < 0.001 vs. TQ+5-FU group; & *p* < 0.05, &&& *p* < 0.001 vs. TQ+5-FU group; $ *p* < 0.05, $$$ *p* < 0.001 vs. HESP+ 5-FU group.

The results of tissue antioxidant/oxidative stress markers (SOD, GSH, CAT, MDA, MPO) are presented in Figure 3A–E. The 5-FU-induced group showed a notable decrease in SOD, GSH, and CAT levels, alongside a significant increase in MPO, MDA, and NO levels compared to the control group. Treatment of TQ, HESP, and their combination with 5-FU significantly restored cardiacSOD, GSH, and CAT levels, and significantly ameliorated MPO, MDA, and NO levels, as compared to the 5-FU group. Once again, the combination therapy showed the most significant improvement, with HESP demonstrating better efficacy compared to TQ when administered individually.

#### 3.2.3. Effect of TQ and HESP on Tissue Inflammatory Markers in Treated Rats

**Figure 4:**
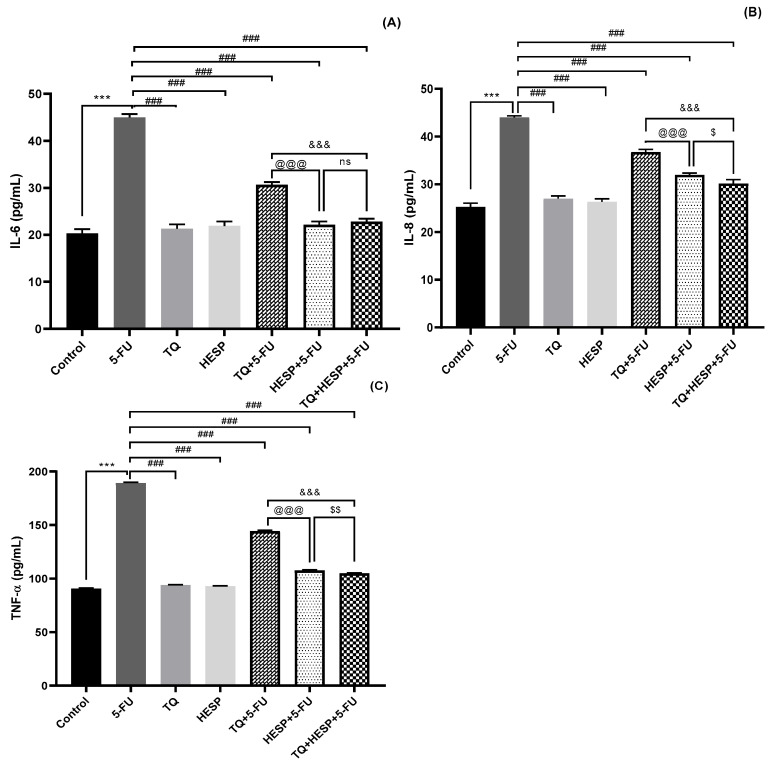
The effect of TQ, HESP, and 5-FU administration on cardiac tissue pro-inflammatory markers and comparison of different groups with each other (one-way Anova followed by Tukey-Kramer multiple comparison test). (**A**) IL-6 level; (**B**) IL-8 level; (**C**) TNF-α level. All results are expressed as the mean ± SD. ns = non-significant; *** *p* < 0.001 vs. control group; ### *p* < 0.001 vs. 5-FU group; @@@ *p* < 0.001 vs. TQ+5-FU group; &&& *p* < 0.001 vs. TQ+5-FU group; $ *p* < 0.05, $$ *p* < 0.01 vs. HESP+ 5-FU group.

The results for tissue inflammatory markers (IL-6, IL-8, TNF-α) are illustrated in Figure 4A–C. The group induced with 5-FU displayed a significant increase in all three inflammation markers compared to the control group. However, upon treating with TQ, HESP, and their combination with 5-FU notably decreased IL-6 levels, IL-8 levels, and TNF-α levels in comparison to the 5-FU group. Notably, the combination group exhibited a significant improvement when compared to the TQ+5-FU and HESP+5-FU groups, with the HESP+5-FU group demonstrating superior outcomes over the TQ+5-FU group.

**Figure 5:**
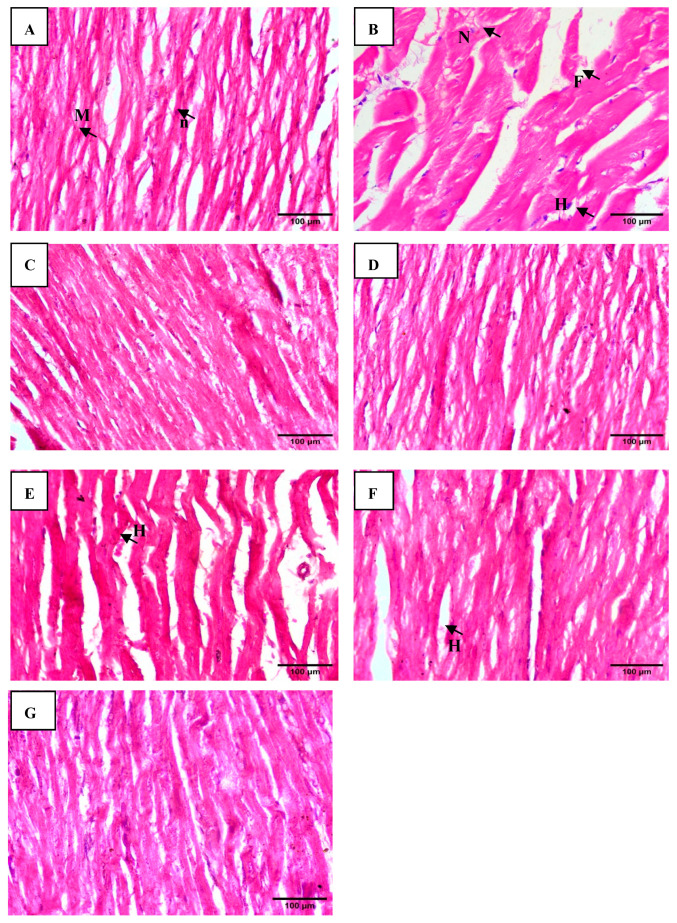
Histopathological findings of cardiac tissues. (**A**) control group showing normal cardiac muscle architecture with striations and myocardial cell membrane integrity. (**B**) 5-FU-treated group showing necrosis of muscle fibers with vacuolar changes evident from fragmentation. (**C**) TQ-treated group showed normal cardiac histology (**D**) HESP-treated group showed normal histology (**E**) TQ+5-FU-treated group showing mild hyperemia. (**F**) HESP+5-FU-treated group showing mild hyperemia. (**G**) TQ+HESP+5-FU-treated group showed almost normal cardiac muscle architecture. M = myocardial fibers; n = nuclei; H = hyperemia; N = necrosis; F = fragmentation; TQ = Thymoquinone; HESP = Hesperidin; 5-FU = 5-Fluorouracil. (H and E; ×400, scale bar = 100 μm).

The histopathological examination of cardiac tissues subjected to different treatments revealed a range of effects. The control group displayed typical cardiac muscle architecture, with intact striations and cell membranes, indicating normal tissue. In contrast, the group treated with 5-FU exhibited severe damage, including necrosis and vacuolar changes in muscle fibers, leading to fragmentation. The TQ- and HESP-treated groups both showed normal cardiac histology. Groups treated with combinations of TQ or HESP with 5-FU showed mild hyperemia. Remarkably, the group treated with a combination of TQ, HESP, and 5-FU showed almost normal cardiac architecture, hinting that TQ and HESP may mitigate the harmful effects of 5-FU, preserving cardiac structure (Figure 5A–G).

## 4. Discussion

5-FU exhibits various toxicities across different organ systems [26]. 5-FU is known to be cardiotoxic, with reported cases of arrhythmias, myocardial infarction, and coronary vasospasm, necessitating prevention and timely intervention for managing these side effects [27]. The use of synthetic compounds for treating side effects can lead to further side effects, prompting a shift towards natural compounds which tend to cause minimum adverse effects. The exact mechanism of how phytoconstituents protect against chemotherapy-induced cardiotoxicity is not clear, however, natural compounds counteract cardiotoxicity by reducing oxidative stress, inhibiting inflammation, easing endoplasmic reticulum stress, modulating apoptosis and autophagy, and enhancing myocardial energy metabolism [28,29,30]. This study explored the cardioprotective potential of TQ and HESP, both independently and together, in mitigating 5-FU-induced cardiotoxicity in Wistar rats, and also examined the binding affinity of these two natural compounds to inflammatory targets. Our study confirmed the cardiotoxic effects of 5-FU, as demonstrated by the significant elevation of cardiac enzymes (CK-MB, CK-NAC, LDH), oxidative stress markers (MDA, MPO), and pro-inflammatory cytokines (IL-6, IL-8, TNF-α) in the 5-FU-treated group. These findings align with the previous literature that has documented similar biochemical alterations in patients and animal models exposed to 5-FU, reinforcing the well-recognized cardiotoxic potential of this chemotherapeutic agent [31,32]. The administration of TQ and HESP was found to significantly ameliorate these adverse effects, as evidenced by a reduction in the levels of cardiac enzymes, pro-inflammatory markers, and oxidative stress markers, along with an increase in antioxidant markers (SOD, GSH, CAT). The individual cardioprotective effects of TQ and HESP have been documented in various studies, which have highlighted their roles in mitigating chemotherapy-induced cardiacdamage through antioxidant and anti-inflammatory mechanisms. Alam et al. (2018) demonstrated that TQ mitigates cardiotoxicity by reducing oxidative stress and inflammation, enhancing antioxidant enzyme activities such as SOD and CAT, and suppressing the release of pro-inflammatory cytokines. Similarly, Alharbiet al. (2023) highlighted the protective role of HESP, showing that it decreases oxidative damage, reduces inflammation by inhibiting pro-inflammatory markers like TNF-α and IL-6, and exhibits antiapoptotic effects, which protect cardiac cells from doxorubicin-induced damage [33,34].

While previous studies have reported the individual effects of TQ and HESP in mitigating chemotherapy-induced organ damage, our study is among the first to comprehensively evaluate their combined effects against 5-FU-induced cardiotoxicity. Our study found that the combination of TQ and HESP provided greater cardio-protection compared to either compound alone. The histopathological analysis corroborated the biochemical findings, where the group treated with both TQ and HESP alongside 5-FU showed nearly normal cardiac architecture, in contrast to the severe necrosis and fragmentation observed in the 5-FU group. These findings suggest that TQ and HESP not only mitigate biochemical markers of cardiotoxicity but also preserve the structural integrity of cardiac tissues. Studies evaluating the protective effects of natural compounds against cardiac damage caused by 5-FU reported similar findings, including reductions in oxidative stress markers, inflammatory markers, and improvements in histopathological parameters [35,36,37]. Onestudy evaluated the protective effects of rutin, Hesperidin alone, and their combination against chemotherapy-induced cardiotoxicity and oxidative stress in male Wistar rats. The combination of rutin and Hesperidin was particularly effective atmitigating cardiac toxicity and atpreserving histological integrity in induced, as well as treated, groups [38]. In our study, the combination likely results in a synergistic effect, where both compounds reduce oxidative damage and inflammation more effectively together than either could alone. This dual action, targeting both oxidative stress and inflammatory cytokines, explains the enhanced cardio-protection observed in the combination therapy, leading to the better preservation of cardiac tissue and function. Chemotherapy and radiotherapy stimulate the production of various pro-inflammatory cytokines, including TNF-α, IL-8, IL-6, IL-10, and TGF-β [39,40].The molecular docking studies in this research were conducted on key inflammatory targets involved in 5-FUinduced cardiotoxicity, including IL-6, IL-8, and TNF-α [41,42]. The docking scores revealed that HESP exhibited stronger binding affinities to these inflammatory proteins than TQ, suggesting that HESP may be more effective atmitigating inflammation. This prediction was confirmed by the in vivo results, where treatment with HESP+5-FU showed superior outcomes in reducing inflammatory markers and improving cardiac function compared to TQ+5-FU. Specifically, HESP’s higher binding affinity correlated with a greater reduction in pro-inflammatory markers, including IL-6, IL-8, and TNF-α in treated rats. A study conducted by Aja et al. (2022) described that HESP violates Lipinski’s Rule of five, however, it showed strong binding affinity towards the compounds studied [43,44]. The purpose of molecular docking is to explore the binding interactions between two molecules, either between proteins or between a protein and a ligand. Following the docking process, the compound was assessed using mathematical models to score its interactions. These scores reflect various chemical properties such as the strength of binding and the energy state, which help in evaluating the potential effectiveness of the compound [45].

Although our findings demonstrate the significant cardioprotective effects of TQ and HESP, there are limitations that should be considered. Firstly, the Wistar rat model, while valuable for preclinical studies, may not fully capture the complexity of 5-FU-induced cardiotoxicity in humans. Secondly, the dosages used in this study may not directly translate to clinical practice, and further research is needed to establish optimal dosing for humans. Future studies should focus on human trials to confirm the efficacy and safety of this combination in clinical settings. Additionally, investigating the long-term safety of TQ and HESP, particularly their effects on other non-target organs, will be essential for translating these findings into practical settings.

## 5. Conclusions

In conclusion, this research has convincingly demonstrated that TQ and HESP offer considerable protective benefits against the cardiotoxic effects of 5-FU. The combined administration of these natural compounds significantly enhanced cardiac function, reduced oxidative stress, and decreased inflammation in treated rats, surpassing the effects observed with individual treatments. Molecular docking studies complemented these findings by illustrating potent interactions between TQ, HESP, and key inflammatory proteins, which likely contribute to their protective mechanisms. This study advances the field by being the first to explore the combined use of TQ and HESP against 5-FU-induced cardiotoxicity. The superior efficacy of this combination, as observed in our in vivo results and supported by molecular docking studies, suggests that a multi-targeted approach can offer more comprehensive cardio-protection. This positions the combination of TQ and HESP as a novel therapeutic strategy for reducing chemotherapy-induced cardiotoxicity. Continued investigation into the clinical applicability of these findings, including optimal dosing and long-term safety evaluations, will be crucial for advancing toward safer chemotherapy regimens. This approach holds potential not only for improving patient compliance but also for extending the therapeutic success of cancer treatments.

## Figures and Tables

**Table 1:** Summary of docking analysis of 1N26, 4XDX, and 2AZ5 with Hesperidin and Thymoquinone.

Proteins	Ligands	Docking Score	Hydrogen Bonding	Polar Interaction	Hydrophobic Interaction
1N26 (IL-6)	Hesperidin	−1.491	ALA127, GLN147, GLU144, ALA160	THR125, GLN147, GLN158, ASN110	ALA127, VAL128, TYR148, PHE155, CYS146, PRO145, LEU159, PHE142, ALA160
	Thymoquinone	−0.780	GLN147	GLN147, GLN158, ASN110	PRO145, CYS157, LEU159, ALA160
4XDX (IL-8)	Hesperidin	−2.367	ASN36, ALA35, PRO53	THR12, ASN36, THR37, SER30	PRO53, VAL27, ILE39, ALA35, CYS34, CYS7, CYS9, ILE10
	Thymoquinone	−1.880	-	ASN36, THR37, THR12	CYS7, CYS9, ILE10, PRO53, LEU51, CYS50
2AZ5 (TNF-α)	Hesperidin	−3.867	ARG259, GLU115	SER116, SER256, SER47, SER43, THR42	ALA257, ALA110, ALA45, ILE111, ILE44, VAL255, PHE49, PRO113
	Thymoquinone	−2.309	-	SER256, SER43, THR42, ASN67	ILE111, PHE112, PHE49, ALA45, ALA257, VAL255

## Data Availability

The data presented in this study are available on request from the corresponding author.
